# Using continuous data on tumour measurements to improve inference in phase II cancer studies

**DOI:** 10.1186/1745-6215-14-S1-O116

**Published:** 2013-11-29

**Authors:** James Wason, Shaun Seaman

**Affiliations:** 1 MRC Biostatistics Unit, Cambridge, UK

## 

In phase II cancer trials, tumour response is either the primary or an important secondary endpoint. Tumour response is a binary composite endpoint determined, according to the RECIST criteria, by 1) whether the percentage change in tumour size is greater than a prescribed threshold and 2) (binary) criteria such as whether a patient develops new lesions.

Further binary criteria, such as death or serious toxicity, may be added to these criteria. The probability of tumour response (i.e. `success' on the composite endpoint) would usually be estimated simply as the proportion of successes among patients. This approach uses the tumour size variable only through a discretised form, viz. whether or not it is above the threshold. In this presentation, we discuss a method which also estimates the probability of success but which gains precision by using the information on the undiscretised (i.e. continuous) tumour size variable. This approach can also be used to increase the power to detect a difference between the probabilities of success under two different treatments in a comparative trial. We demonstrate these increases in precision and power using simulated data.

We also apply the method to real data from a phase II cancer trial, and show that it results in a considerably narrower confidence interval for the probability of tumour response. Extending the work to other endpoints used in phase II trials, such as progression-free survival will be discussed.

